# Targeting Photoaging With Heat-Killed Escherichia coli Nissle 1917: A Novel Cellular Model and Anti-photoaging Strategy

**DOI:** 10.7759/cureus.91268

**Published:** 2025-08-29

**Authors:** Farshid Zandsalimi, Zahra Azizi, Mohammad Ali Mazloomi, Mansoreh Abdolhosseini, Moloud Absalan, Mobina Tabibian, Elahe Motevaseli

**Affiliations:** 1 Department of Molecular Medicine, School of Advanced Technologies in Medicine, Tehran University of Medical Sciences, Tehran, IRN; 2 Department of Medical Biotechnology, School of Advanced Technologies in Medicine, Tehran University of Medical Sciences, Tehran, IRN; 3 Department of Cellular and Molecular Biology, Faculty of Life Sciences and Biotechnologies, Shahid Beheshti University of Medical Sciences, Tehran, IRN

**Keywords:** e. coli nissle 1917 (ecn), photoaging, senescence, skin, ultraviolet (uv) radiation

## Abstract

Background: Ultraviolet (UV) radiation is a major contributor to skin aging, manifesting as wrinkles, pigmentation, and structural dysfunction, collectively termed photoaging. Chronic photoaging is strongly linked to an increased risk of skin cancer. Despite the growing demand for effective anti-photoaging agents, a gap remains in both understanding the molecular basis of photoaging and validating the efficacy of new therapeutic candidates. This study aimed to establish a reliable in vitro photoaging model and evaluate the potential anti-photoaging effects of heat-killed *Escherichia coli* Nissle 1917 (EcN).

Methods: Human dermal fibroblast (HDF) cells were exposed to UVB radiation three times at one-day intervals. Cell viability was assessed using the MTT assay. Post-irradiation, cells were treated with varying concentrations of heat-killed EcN. Effects were evaluated through live/dead staining, apoptosis assay, cell cycle analysis, and reactive oxygen species (ROS) quantification. Resveratrol (RES), a known anti-inflammatory and anti-aging compound, was used as a reference control.

Results: UVB exposure at 0.3 J/cm² reduced HDF cell viability to approximately 50% compared to the control group. Morphological and biochemical assessments validated the photoaging model’s reliability. Treatment with 10% v/v heat-killed EcN and 100 μM RES after each UVB cycle significantly restored cell viability, reduced apoptosis and cell cycle arrest, and markedly decreased intracellular ROS levels.

Conclusion: This study demonstrates the efficacy of heat-killed EcN and RES in alleviating UVB-induced photoaging, likely via suppression of oxidative stress and apoptosis. The developed in vitro model offers a robust platform for future investigations into molecular mechanisms of photoaging and the evaluation of emerging photoprotective agents.

## Introduction

The skin, being continuously exposed to sunlight throughout life, is significantly affected by ultraviolet (UV) radiation. Prolonged and unprotected exposure is a major contributor to premature skin aging, characterized by visible signs such as wrinkles, loss of firmness, and pigmentation, commonly referred to as photoaging [1]. Beyond its aesthetic implications, chronic photoaging can predispose individuals to skin cancer.

UV radiation is categorized into three main bands: UVC (100-280 nm), UVB (280-315 nm), and UVA (315-400 nm). UVC, the shortest and most energetic band, is effectively absorbed by the Earth’s atmosphere and thus can be disregarded [2]. UVA, comprising the largest portion of solar UV radiation, has the lowest energy and consequently the least damaging biological effects. UVB, situated in the middle of the spectrum, is the most biologically active and is chiefly responsible for cellular damage, photoaging, and UV-induced carcinogenesis [3].

As the body’s primary barrier against UV radiation, skin performs a critical defensive role. However, its structure and function decline with age and can be further compromised by repeated or prolonged UV exposure. Over time, numerous synthetic and naturally derived agents have been explored to mitigate UV-related damage, an area of growing interest in both cosmetic and medical science. However, despite these efforts, no fully effective strategy has been established to prevent photoaging. The complexity of its molecular underpinnings and the lack of robust experimental models remain key limitations in advancing this field.

Probiotics, defined as live microorganisms that confer health benefits to the host, have a long history of human use [4]. Advances in probiotic research and industrial biotechnology have broadened their applications and commercial availability [5]. Nonetheless, the viability of live organisms in probiotic formulations remains critically low, and safety concerns persist, particularly regarding their use on compromised skin surfaces where microbial translocation to the bloodstream is possible [6]. Postbiotics - non-viable microorganisms or their metabolic products - have emerged as a safer alternative, offering probiotic-like benefits without the associated risks of live-cell applications [7]. Accumulating evidence suggests that postbiotics can modulate immune responses and reproduce probiotic functionality in a more controlled manner, even in immunocompromised individuals [8].

This study investigates the anti-photoaging potential of inactivated *Escherichia coli *Nissle 1917 (EcN) using an in vitro model. EcN is a well-characterized, non-pathogenic probiotic strain known for its anti-inflammatory effects, particularly in the context of inflammatory bowel disease (IBD), where its clinical efficacy has been widely studied [9]. However, the effects of its inactivated form remain underexplored, especially in relation to skin photoaging. To the best of our knowledge, this is the first study to examine the photoaging-modulating properties of inactivated EcN. A robust photoaging model was first established using human dermal fibroblast (HDF) cells, validated by cellular and molecular analyses. The effects of inactivated EcN were then assessed within this model. In addition to providing evidence for the therapeutic potential of postbiotic EcN, the study introduces a reliable In vitro system for investigating skin photoaging and sets the stage for future research.

## Materials and methods

Materials

Dulbecco’s modified eagle medium (DMEM), fetal bovine serum (FBS), penicillin-streptomycin (Pen-Strep), phosphate-buffered saline (PBS), and trypsin were procured from Biowest (France). MTT reagent (3-[4,5-dimethylthiazol-2-yl]-2,5-diphenyltetrazolium bromide), dimethyl sulfoxide (DMSO), glycerol, 0.22 μm membrane filters, Luria-Bertani (LB) broth, Annexin V, propidium iodide (PI), resveratrol (RES), acetone, and DCFH-DA (2′,7′-dichlorodihydrofluorescein diacetate) were purchased from Sigma-Aldrich (Germany).

Cell culture

HDF cells were obtained from the Iranian Biological Resource Center (IBRC), Tehran, Iran. Cells were cultured in DMEM supplemented with 10% FBS and 1% Pen-Strep. Upon reaching 90% confluency, cells were harvested using trypsinization and subsequently passaged for use in downstream experiments.

UV radiation

HDF cells (1.2 × 10⁵ cells per well) were seeded into six-well culture plates. Once the cultures reached approximately 90% confluency, the culture medium was removed, and cells were washed and covered with a thin layer of PBS. Cells were then exposed to UVB radiation at graded intensities (0.1, 0.2, 0.3, 0.4, 0.5, and 0.6 J/cm²). Following irradiation, fresh complete medium was added to each well, and plates were returned to the incubator. This irradiation protocol was repeated three times for each intensity at 24-hour intervals. This exposure pattern was chosen based on prior studies demonstrating that repeated UVB irradiation induces more robust photoaging markers in fibroblasts than single-dose protocols [10,11]. UV exposure was carried out using the BioSun UV irradiation system (Vilber Lourmat, France).

Cell viability assay

 Cell viability was assessed using the standard MTT assay [12]. Briefly, following completion of the UVB irradiation protocol, culture medium was removed from each well, and MTT reagent was added at a final concentration of 0.5 mg/mL. After a four-hour incubation period, DMSO was introduced to solubilize the formazan crystals. Absorbance was measured at 570 nm, with a reference wavelength of 690 nm, using a Cytation™ microplate reader (BioTek, USA). Viability was expressed as a percentage relative to the non-irradiated control group. The UVB intensity that reduced cell viability to approximately 50% was selected as the experimental dose for subsequent analyses.

Inactivation of *E. coli* Nissle 1917 (EcN)

An overnight culture of EcN was grown in LB broth and subsequently centrifuged at 8000 × g for 10 minutes. The resulting pellet was washed three times with sterile PBS. A suspension containing 1 × 10⁸ colony-forming units (CFU)/mL was then subjected to thermal inactivation by incubation at 70 °C for one hour in a water bath. Successful inactivation of EcN was confirmed by inoculating the heated suspension on LB agar plates and verifying the absence of bacterial growth.

Effect of heat-killed EcN on irradiated HDF cells

To evaluate the protective effect of heat-killed EcN on UVB-irradiated HDF cells, cell viability was assessed using the standard MTT assay. Briefly, 8 × 10³ HDF cells per well were seeded into 96-well plates. Upon reaching approximately 90% confluency, cells were subjected to UVB irradiation. Immediately following exposure, fresh complete culture medium supplemented with different concentrations of inactivated EcN (5, 10, 20, 30, 40, and 50% v/v of final volume) was added to each well. Viability was then measured as previously described using the MTT assay. The concentration of heat-killed EcN that conferred the highest protective effect on irradiated cells was selected for subsequent experiments.

Effect of RES on irradiated HDF cells

To validate the responsiveness of the In vitro photoaging model, the effect of RES - a natural polyphenolic compound with notable anti-inflammatory properties - was evaluated in irradiated HDF cells. Previous studies have shown that RES can reduce photoaging markers and protect fibroblasts against UV-induced damage [13]. In this study, the standard MTT assay was employed to assess cell viability. HDF cells (8 × 10³ cells per well) were seeded into 96-well plates and, upon reaching ~90% confluency, subjected to UVB irradiation. Following each irradiation cycle, the culture medium supplemented with varying concentrations of RES (5, 10, 20, 40, 60, and 100 μM) was added to the cells. Cell viability was measured at the end of the treatment period as previously described using the MTT assay.

Live and dead analysis

To evaluate the impact of UVB irradiation and the selected treatments on cell viability, PI fluorescent staining was performed. HDF cells (1.2 × 10⁵ cells per well) were seeded into six-well plates and subjected to the UVB irradiation protocol. Following each irradiation cycle, cells were treated with the optimal concentrations of heat-killed EcN and RES, as determined in previous assays. After completion of the treatment cycles, cells were harvested and stained with PI solution to label non-viable cells. The proportion of live versus dead cells was then quantified via flow cytometric analysis.

Apoptosis assay

Apoptotic cell death in HDF cells was evaluated using Annexin V-FITC/PI dual staining. Briefly, HDF cells were seeded in six-well plates at a density of 1.2 × 10⁵ cells per well. Following the completion of the UVB irradiation and treatment cycles, cells were harvested by trypsinization. Staining was performed by resuspending the collected cells in 1x binding buffer and adding Annexin V-FITC and PI according to the manufacturer’s protocol. After a 15-minute incubation in the dark at room temperature, samples were analyzed using a FACS Calibur flow cytometer (BD Biosciences, San Jose, USA).

Cell cycle analysis

To evaluate the distribution of cell cycle phases (G₀/G₁, S, and G₂/M), HDF cells were subjected to UVB irradiation and treatment protocols as previously described. After the final cycle, cells were harvested and incubated for 30 minutes with a standard staining solution containing PI, DNase-free RNase, and PBS. DNA content was then analyzed using a BD FACS Calibur flow cytometer (BD Biosciences, San Jose, USA), and the proportion of cells in each cell cycle phase was determined.

Reactive oxygen species (ROS) measurement

Intracellular reactive oxygen species (ROS) levels in HDF cells were quantified using DCFH-DA staining followed by flow cytometric analysis. Irradiated and treated cells were prepared and harvested as previously described. Cells were then incubated with a solution containing PI and DCFH-DA for 20 minutes at room temperature, protected from light. Fluorescence intensity-indicative of ROS levels-was measured using a BD FACS Calibur flow cytometer (BD Biosciences, San Jose, USA).

Statistical analysis

All experiments were conducted in triplicate, and data are presented as mean ± standard deviation. Differences between experimental groups were assessed using two-way analysis of variance (ANOVA), followed by Tukey’s post-hoc multiple comparison test. A p-value < 0.05 was considered statistically significant. Graphs were generated using GraphPad Prism 8 (GraphPad Software, LLC).

## Results

Construction of the model

**Figure 1:**
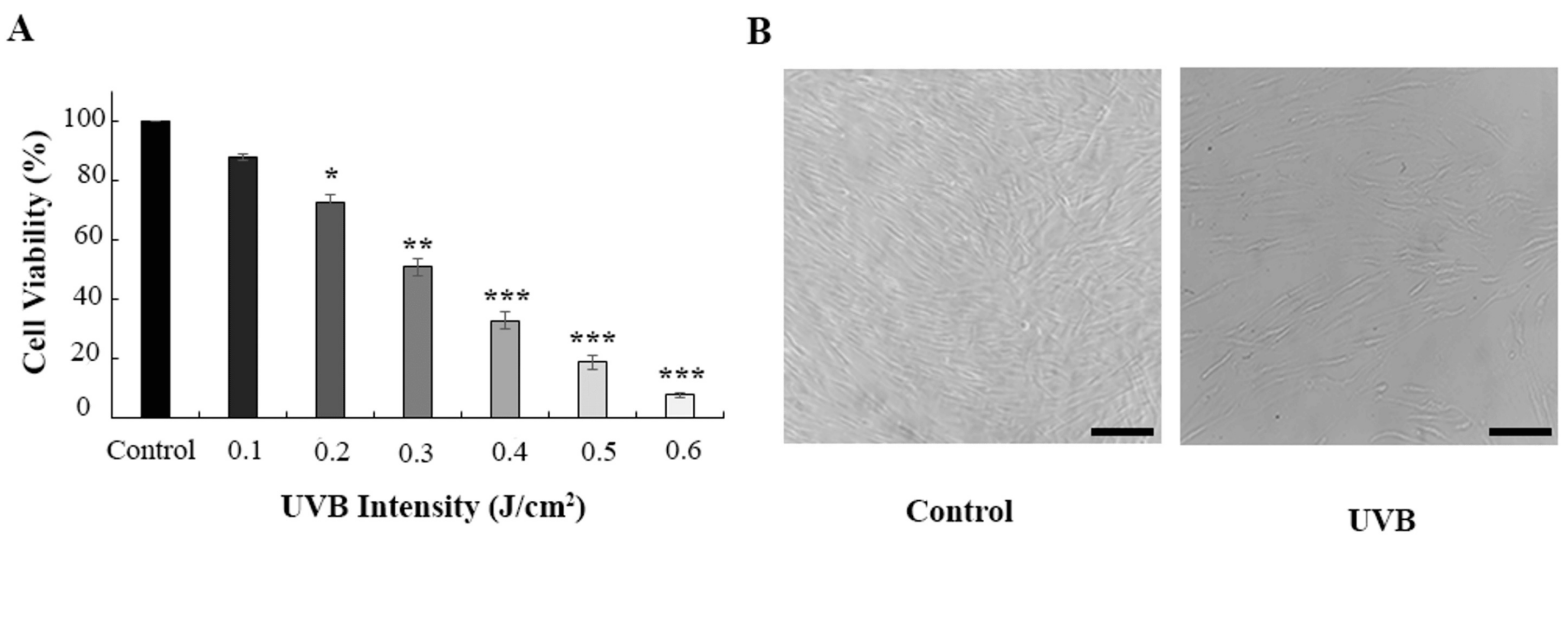
Effect of UVB on HDF cells. A) The viability of the cells decreased in a dose-dependent manner. B) 0.3 J/cm^2^ of UVB radiation induced significant morphology changes in HDF. * shows significance level compared to the control group (*p < 0.05, **p < 0.01, and ***p < 0.001). HDF: human dermal fibroblast

To establish a reliable In vitro photoaging model, HDF cells were exposed to escalating doses of UVB radiation. As shown in Figure 1A, increasing UVB intensities corresponded to a progressive decline in cell viability. Specifically, exposure to 0.3 J/cm² UVB reduced cell viability to 52.14%, and this dose was subsequently selected for downstream experiments. Morphological assessment of HDF cells following three repeated exposures to 0.3 J/cm² UVB revealed pronounced cellular alterations. Compared to normal fibroblasts, which exhibited a spindle-shaped morphology with clear cell-cell boundaries-irradiated cells showed signs of atrophy, swelling, disrupted intercellular contact, and elevated cell debris. These morphological changes are consistent with the hallmark features of photoaged fibroblasts, as illustrated in Figure 1B.

Effect of heat-killed EcN on irradiated HDF cells

**Figure 2:**
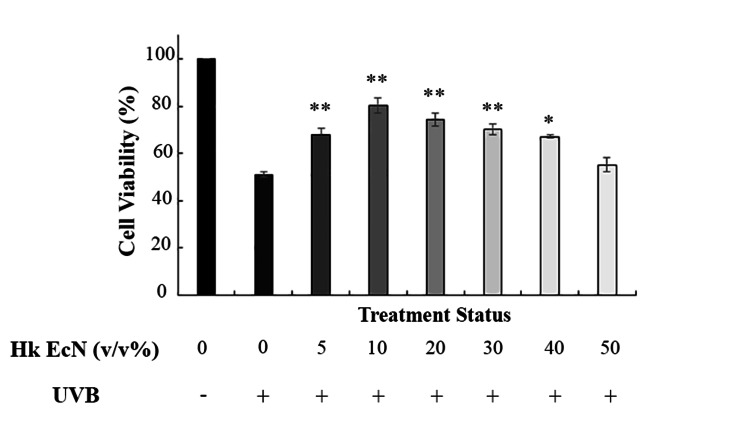
Effect of different concentrations of heat-killed EcN on the viability of irradiated HDF cells. 10% v/v had the maximum protective effect. * shows significance level compared to the control group (*p < 0.05, **p < 0.01, and ***p < 0.001). Hk EcN: heat-killed *Escherichia coli *Nissle 1917, HDF: human dermal fibroblast

To assess the protective effects of heat-killed EcN on UVB-irradiated HDF cells, various concentrations of the extract were introduced into the culture medium following each irradiation cycle. Cell viability was then evaluated using the MTT assay. As illustrated in Figure 2, heat-killed EcN significantly restored cell viability across all tested concentrations. Notably, treatment with 10% v/v EcN yielded the highest protective effect, elevating cell viability from 51% in the UVB group to 80.21%. Based on this finding, the 10% concentration was selected for use in subsequent experiments.

Effect of RES on irradiated HDF cells

**Figure 3:**
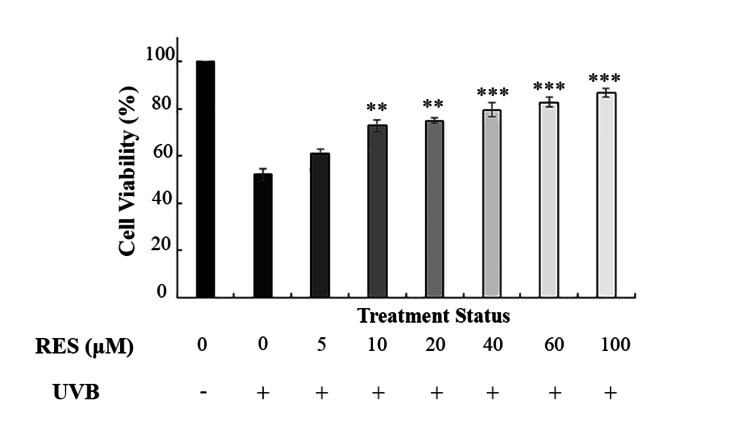
Effect of different concentrations of RES on the viability of irradiated HDF cells. RES restored the viability of irradiated HDF cells in a dose-dependent manner. * shows significance level compared to the control group (*p < 0.05, **p < 0.01, and ***p < 0.001). RES: resveratrol, HDF: human dermal fibroblast

The protective effect of RES, a well-characterized anti-inflammatory polyphenol, was evaluated in UVB-irradiated HDF cells using the MTT assay. As shown in Figure 3, RES treatment effectively mitigated UVB-induced cytotoxicity, with all tested concentrations resulting in enhanced cell viability. This protective response exhibited a concentration-dependent pattern. Treatment with 100 μM RES demonstrated the highest efficacy, increasing cell viability from 51% (UVB-only group) to 86.83%. Accordingly, this concentration was selected for subsequent experiments.

Live and dead analysis

**Figure 4:**
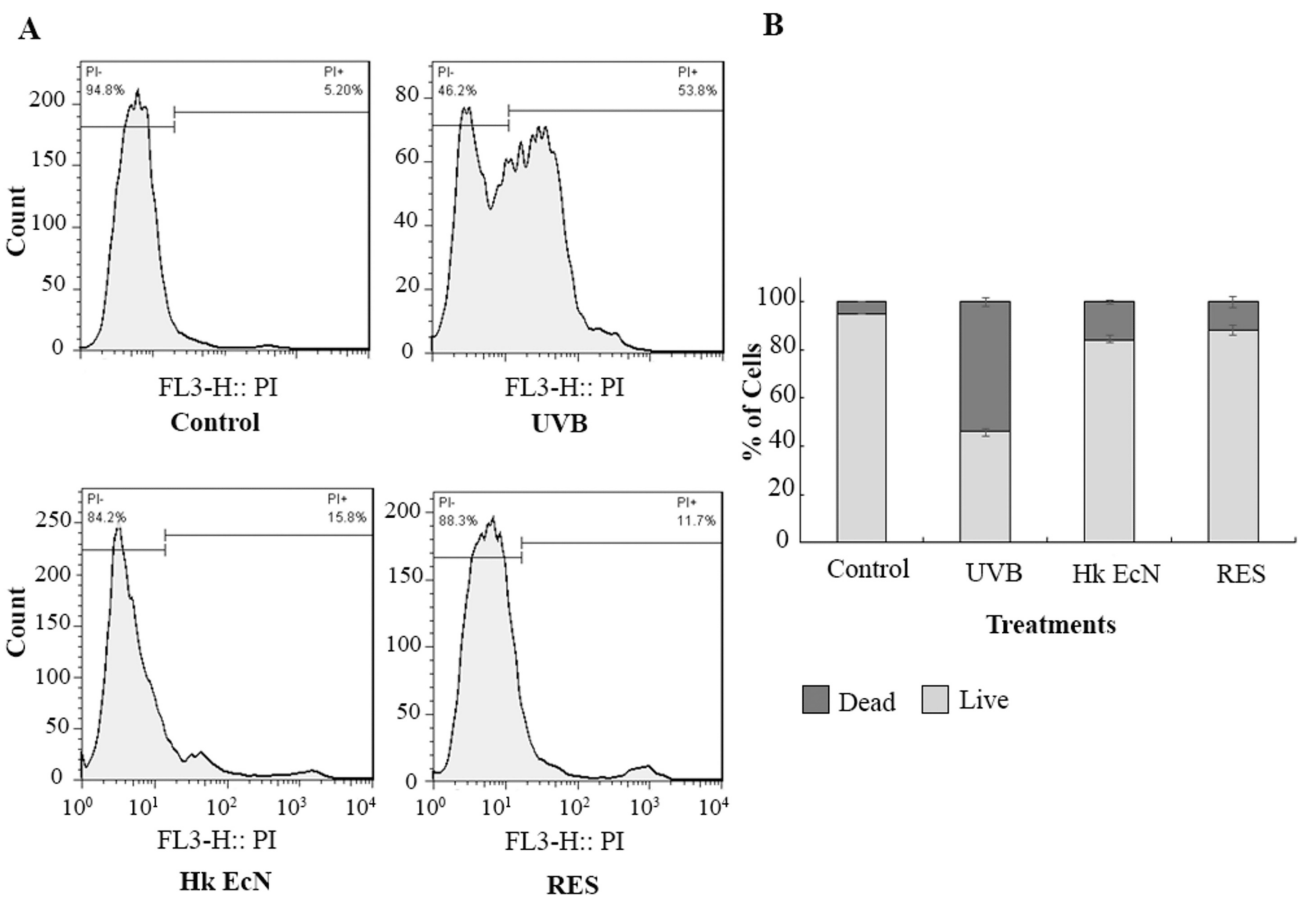
Live and dead analysis using PI staining. A) Flow cytometry plots. B) Quantitative comparison of live and dead cells in the studied groups. Control: HDF cells, UVB: irradiated HDF cells (0.3 J/cm2), Hk EcN: irradiated HDF cells treated with 10% v/v heat-killed *E. coli *Nissle 1917, and RES: irradiated HDF cells treated with 100 μM of resveratrol. PI: propidium iodide, HDF: human dermal fibroblast, EcN: *Escherichia coli *Nissle 1917

To compare the protective effects of heat-killed EcN and RES on UVB-irradiated HDF cells, PI staining was performed. Following each UVB irradiation cycle, cells were treated with fresh medium containing 10% v/v heat-killed EcN or 100 μM RES. At the conclusion of the radiation protocol, the proportion of live and dead cells was quantified. As shown in Figure 4, UVB irradiation effectively induced photoaging, reducing cell viability to 46.2%. However, treatment with heat-killed EcN and RES significantly attenuated UVB-induced cytotoxicity, restoring the proportion of viable cells to 88.3% and 84.2%, respectively.

Apoptosis assay

**Figure 5:**
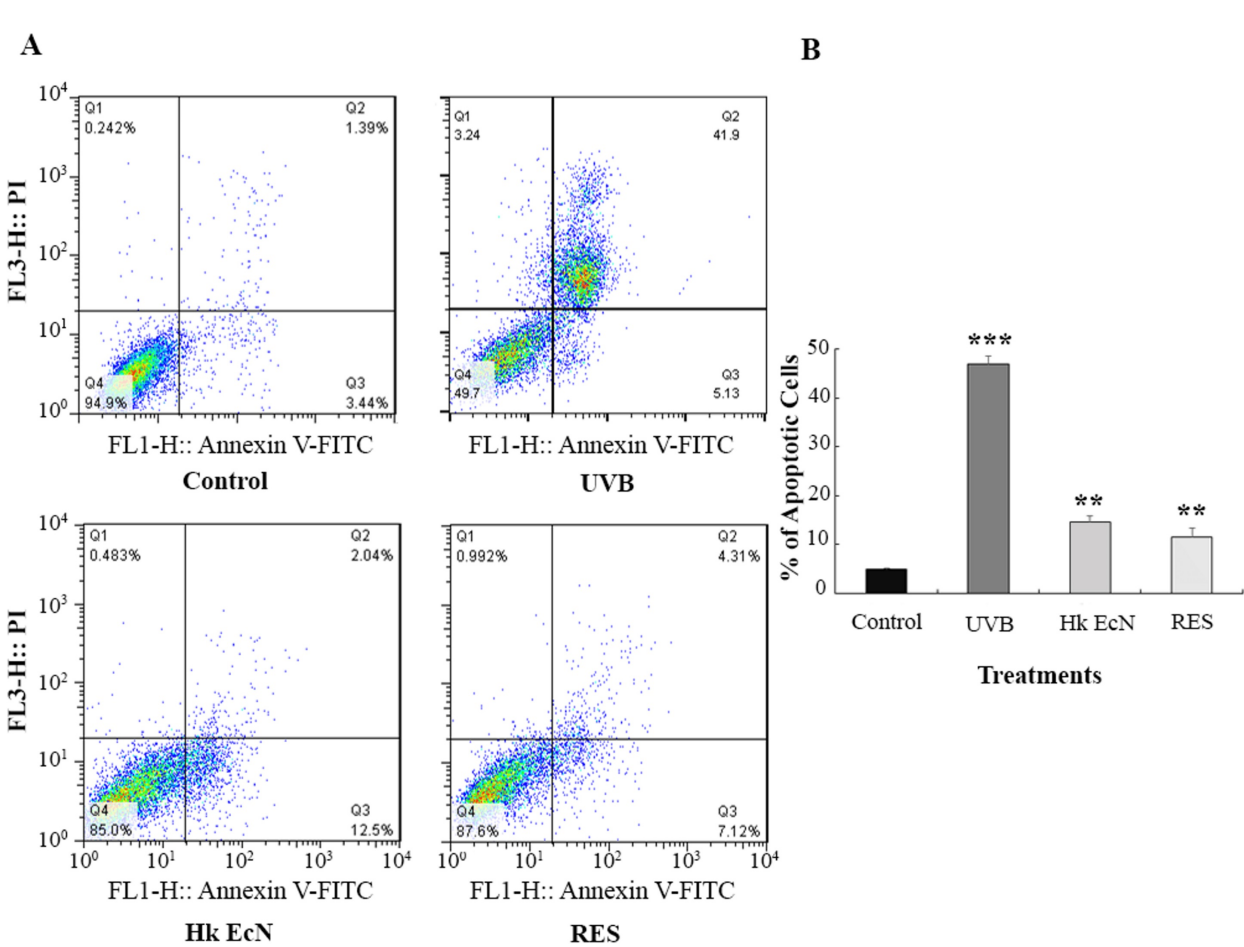
Apoptosis assay. A) Flow cytometry plots. B) Quantitative comparison of the studied groups. * shows significance level compared to the control group (*p < 0.05, **p < 0.01, and ***p < 0.001). Control: HDF cells, UVB: irradiated HDF cells (0.3 J/cm2), Hk EcN: irradiated HDF cells treated with 10% v/v heat-killed *E. coli *Nissle 1917, and RES: irradiated HDF cells treated with 100 μM of resveratrol. HDF: human dermal fibroblast, EcN: *Escherichia coli *Nissle 1917

To evaluate the capacity of the developed photoaging model to induce apoptosis and the potential protective effects of heat-killed EcN and RES, HDF cells were analyzed using Annexin V-FITC/PI staining. UVB irradiation alone induced substantial apoptosis in HDF cells, with a combined early and late apoptotic rate of 47%. Necrotic cell death was also elevated, reaching 3.24%. Treatment with 10% v/v heat-killed EcN and 100 μM RES after each UVB exposure significantly reduced apoptosis. Apoptotic rates decreased to 14.5% and 11.4% in EcN- and RES-treated groups, respectively. Furthermore, necrosis was markedly suppressed in both treatment conditions, dropping to 0.99% for EcN and 0.48% for RES (Figure 5).

Cell cycle analysis

**Figure 6:**
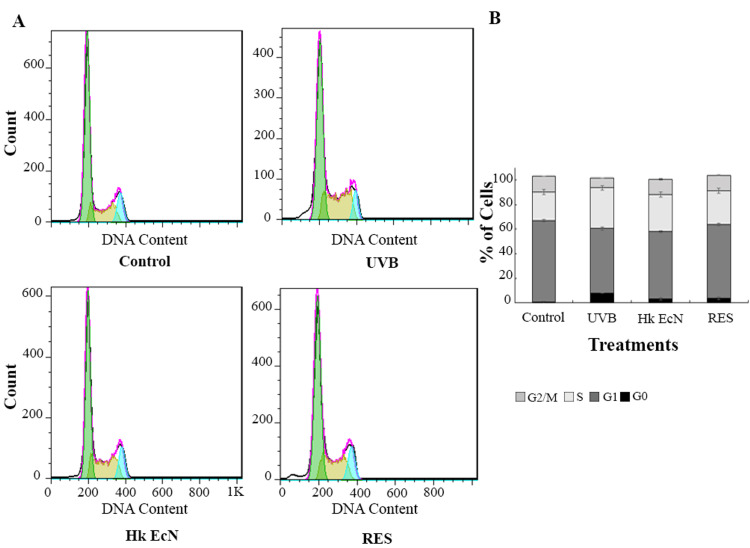
Cell cycle analysis. A) Flow cytometry plots. B) Quantitative comparison of cell cycle phases, including G0/G1, S, and G2/M, in the studied groups. Control: HDF cells, UVB: irradiated HDF cells (0.3 J/cm2), Hk EcN: irradiated HDF cells treated with 10% v/v heat-killed *E. coli *Nissle 1917, and RES: irradiated HDF cells treated with 100 μM of resveratrol. HDF: human dermal fibroblast, EcN: *Escherichia coli* Nissle 1917

Cell cycle distribution across experimental groups was analyzed and compared, as shown in Figure 6. UVB irradiation caused marked disruptions in HDF cell cycle progression, evidenced by a decrease in the G₂/M phase population (7.96% vs. 13.05% in control) and a substantial increase in the sub-G₁ population (7.76% vs. 0.51% in control), indicating UVB-induced cell cycle arrest and apoptotic activity. Treatment with heat-killed EcN conferred partial protective effects, notably reducing the sub-G₁ population to 3.19% and restoring G₂/M levels to 12.36%, suggesting alleviation of DNA damage and promotion of cell cycle recovery. RES treatment demonstrated a distinct protective pattern, characterized by enhanced G₁ phase retention (59.94%) and normalized G₂/M phase distribution (12.52%), supporting its regulatory influence on cell cycle dynamics.

Reactive oxygen species (ROS) measurement

**Figure 7:**
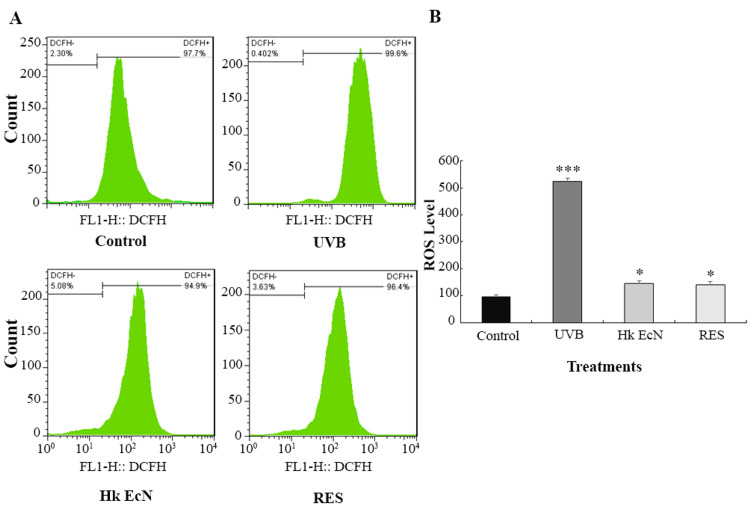
ROS level measurement. A) Flow cytometry plots. B) Quantitative comparison of ROS levels in the studied groups. * shows significance level compared to the control group (*p < 0.05, **p < 0.01, and ***p < 0.001). Control: HDF cells, UVB: irradiated HDF cells (0.3 J/cm2), Hk EcN: irradiated HDF cells treated with 10% v/v heat-killed *E. coli *Nissle 1917, and RES: irradiated HDF cells treated with 100 μM of resveratrol. ROS: reactive oxygen species, HDF: human dermal fibroblast, EcN: Escherichia coli Nissle 1917

Intracellular ROS levels in HDF cells were quantified using DCFH-DA staining, followed by flow cytometric analysis. Compared to the non-irradiated control group (ROS level: 96.7), UVB irradiation significantly elevated ROS production, reaching 524 arbitrary units. Treatment with 10% v/v heat-killed EcN and 100 μM RES markedly reduced ROS accumulation, with levels dropping to 145 and 141, respectively, indicating substantial antioxidant and cytoprotective effects (Figure 7).

## Discussion

A reliable in vitro model of photoaging was established in this study. We showed that 0.3 J/cm² of UV-B radiation would decrease the viability of fibroblast cells to 50% of normal. The induction of photoaging in the model was confirmed by microscopy appraisal, as well as live and dead staining, apoptosis assay, cell cycle analysis, and ROS measurement. Functional analysis of the established model also revealed that the treatment of fibroblast cells with 10% v/v heat-killed EcN and 100 μM RES after each UVB cycle significantly restored cell viability, reduced apoptosis and cell cycle arrest, and markedly decreased intracellular ROS levels.

UV radiation remains the most prevalent and detrimental external threat to skin health. While moderate exposure to UV contributes to beneficial physiological processes such as vitamin D synthesis and endorphin production, excessive irradiation is a major risk factor for skin disorders, including cancer [14]. The skin's multilayered architecture-comprising the epidermis, dermis, and hypodermis, evolved to limit UV penetration. Resident cells such as fibroblasts, keratinocytes, and Langerhans cells play essential roles in neutralizing UV-induced damage [15]. Nonetheless, these defenses can be overwhelmed by genetic susceptibilities and cumulative UV exposure, leading to photoaging and related conditions [16].

Over recent decades, a wide array of anti-UV agents has been developed to chemically, physically, or synergistically mitigate UV damage [17]. However, innovation in this field is hampered by the absence of a robust model system for evaluating anti-photoaging efficacy. Ethical concerns restrict the use of animal models [18], while existing in vitro models often suffer from inconsistency and limited predictive power.

This study aimed to construct a reliable In vitro photoaging model using HDF cells subjected to varying doses of UVB radiation. Fibroblasts play a vital role in skin integrity through their secretion of extracellular matrix (ECM) components, such as collagen and elastin, as well as their involvement in inflammation via cytokine and chemokine production. Cellular senescence or UV-induced damage can severely impair these functions, contributing to photoaging [19]. UVB radiation is a principal driver of photoaging, causing DNA damage, ECM degradation, and chronic inflammation, all of which are linked to skin aging and carcinogenesis. UVB exposure activates signaling pathways such as p53, NF-κB, and AP-1, which promote fibroblast senescence, inhibit proliferation, and remodel ECM architecture [20]. In this study, repeated exposure to UVB at 0.3 J/cm² over three cycles effectively induced photoaging in HDF cells, reducing viability to nearly 50% of control levels.

The established model was then used to evaluate the anti-photoaging potential of heat-killed EcN and RES. Both treatments demonstrated significant protective effects, as evidenced by PI staining and flow cytometry, which revealed reduced cytotoxicity following UVB exposure. Apoptosis and cell cycle analyses indicated that EcN and RES mitigated UVB-induced damage by suppressing apoptosis and alleviating cell cycle arrest. UVB-induced apoptosis proceeds via both intrinsic and extrinsic pathways, commonly initiated by DNA damage and activation of p53-regulated pro-apoptotic genes including BAX, PUMA, and NOXA [21,22]. While previous studies focused on apoptotic pathways, our findings suggest that UVB can also trigger necrosis in HDF cells - a phenomenon previously observed primarily in lymphocytes exposed to different UVB doses [23]. This insight expands current understanding of dose-dependent cell death mechanisms in fibroblasts and highlights the versatility of the developed model for future mechanistic studies.

The anti-apoptotic effects of EcN and RES appear to be linked to their ability to reduce intracellular ROS. UVB exposure led to a pronounced accumulation of ROS, which regulates a network of signaling cascades that drive senescence and cell death. For example, ROS-mediated activation of MAPK signaling increases AP-1 activity through ERK and p38 pathways, thereby enhancing MMP expression and collagen degradation - hallmarks of photoaging [24]. ROS also exacerbates oxidative stress, damages cellular structures, and modulates apoptotic gene expression by influencing p53, Bcl-2 family proteins, and IAPs [21,25].

The antioxidant capacity of EcN and RES has been previously reported. EcN's therapeutic potential in IBD is attributed to its ROS-scavenging activities via antioxidant enzymes such as superoxide dismutase (SOD) and catalase (CAT) [26]. Surface components like exopolysaccharides (EPS) and probiotic-derived factors further enhance this activity [27]. The anti-photoaging of heat-killed probiotics has been explored in other species. For instance, it was shown that heat-killed *Lactobacillus rhamnosus* ATCC 7469 could protect mouse skin fibroblast cells from UVB radiation and significantly increase their viability. In that study, the heat-killed *L. rhamnosus* ATCC 7469 was added pre-exposure to UVB. They improved the radiated cells' radical-scavenging potency by activating SOD2 and inducing Nrf2 and Sirt3 proteins' expression [28]. RES's antioxidant effects, on the other hand, are due to the direct reaction of this component with free radicals like hydroxyl and superoxide. RES could also effectively modulate signaling pathways like NF-κB to neutralize free radical species [29].

Limitations of the study

This study mainly focused on the development of an In vitro model to assess the anti-photoaging property of elements. The primary function of the model was also investigated using heat-killed EcN. While this objective and the presented findings valuably provide a basis for future studies, there are some limitations that should be considered. The signaling pathways affected by UVB radiation and the impact of treatments on these pathways were not appraised. Such an investigation could be seen as an extra authenticator of the function of the photoaging model. More molecular assessments could also expand our knowledge about UVB-skin interaction and involved mechanisms. In addition, here we did not investigate the mechanisms underlying the anti-photaging effect of heat-killed EcN on UV-B irradiated fibroblast cells. Furthermore, we did not try to develop a clinical approach for topical applications of heat-killed EcN. Although finding ways to formulate and clinical applications of heat-killed EcN was out of the scope of this study, this is a critical step in the commercialization of this product. In sum, subsequent detailed studies on the suggested model and clinical utilization of the proposed treatments are planned and strongly encouraged.

## Conclusions

This study establishes a robust in vitro model for evaluating the anti-photoaging properties of active compounds in HDF cells. The model’s performance was validated through a series of morphological, biochemical, and molecular assessments. Using this platform, heat-killed EcN and RES demonstrated notable anti-photoaging activity by suppressing UVB-induced apoptosis and reducing intracellular ROS levels. These findings provide a foundation for future investigations aimed at assessing additional bioactive compounds with photoprotective potential. Moreover, the development of molecular markers to further characterize photoaging responses represents a promising direction for expanding the applicability of this model in dermatological research and therapeutic development.
